# Multiple sclerosis in sarcoidosis patients: Two case reports

**DOI:** 10.1002/ccr3.6332

**Published:** 2022-09-12

**Authors:** Masoud Etemadifar, Armin Mehri, Nahad Sedaghat, Mehri Salari, Parsa Tavassoli Naini

**Affiliations:** ^1^ Neurosurgery Research Department Alzahra University Hospital Isfahan University of Medical Sciences Isfahan Iran; ^2^ Functional Neurosurgery Research Center Shohada Tajrish Comprehensive Neurosurgical Center of Excellence Shahid Beheshti University of Medical Sciences Tehran Iran

**Keywords:** case report, multiple sclerosis, neurosarcoidosis, sarcoidosis

## Abstract

Two cases of sarcoidosis referred to our clinic with neurological symptoms. They were diagnosed with multiple sclerosis using non‐invasive studies. The first patient refused treatment and died of myocardial infarction 6 months after visiting our clinic. The second received interferon‐beta and methotrexate with a favorable outcome after 3 years. Since the possible similar presentation of the two conditions could appear indistinct for certain diagnosis, accurate evaluation of symptoms and paraclinical data can provide the best approach to each condition.

## 1 INTRODUCTION

Sarcoidosis is a multisystem granulomatous disease of unknown etiology that mostly affects lymphatics, lungs, eyes, and skin, but can involve almost any organ or tissue.^1^ Studies suggest that sarcoidosis develops as a result of genetic susceptibility and as‐yet unknown environmental triggers.^2^, ^3^, ^4^ Almost all of the sarcoidosis patients have either intrathoracic lymph‐node enlargement, pulmonary involvement, skin or ocular signs/symptoms, or a combination of these findings.^5^, ^6^ Diagnosis is based on clinical and radiological findings, observation of non‐caseating granulomas in histological study of one or more tissues, and importantly, exclusion of other entities.^7^ Sarcoidosis can affect the central and/or peripheral nervous systems—a condition referred to as neurosarcoidosis (NS). Although rare, neurologic involvement occurs in 5%–15% of all sarcoidosis patients. In affected cases, NS usually causes severe and permanent disability.^8^, ^9^

Multiple sclerosis (MS)—an autoimmune demyelinating entity of the CNS—is a particularly challenging differential diagnosis of NS. That is mainly because patients with sarcoidosis and MS are of similar ages and may present with overlapping clinical features such as damage to cranial nerves, myelopathy, and demyelinating lesions of the CNS.^10^, ^11^ Meanwhile, an inaccurate diagnosis would not only prevent the patients from receiving disease‐modifying therapies (DMTs) for their MS, but also may cause their exposure the to some NS medications that may worsen MS—for example, tumor necrosis factor alpha antagonists.^12^, ^13^, ^14^ Meanwhile, although CNS histological studies are considered gold standard for differentiation of NS from other conditions, nervous system biopsies are unfeasible, invasive, and potentially dangerous procedures; hence, diagnosing NS based on a combination of clinical findings, CSF analysis, imaging, and presence of typical non‐caseating granulomas in extra‐nervous tissues is also considered acceptable.^15^, ^16^ Consequently, diagnosing and treating coexisting MS in sarcoidosis patients presenting with neurological illnesses seems puzzling. Thus, aiming to share our experience with this rare phenomenon, we described two patients with known sarcoidosis, who presented with neurological manifestations to our clinic. Our diagnostic and treatment approach along with previous studies could guide clinicians in the future to identify and manage such rare events properly.

## 2 CASE PRESENTATION 1

**FIGURE 1:**
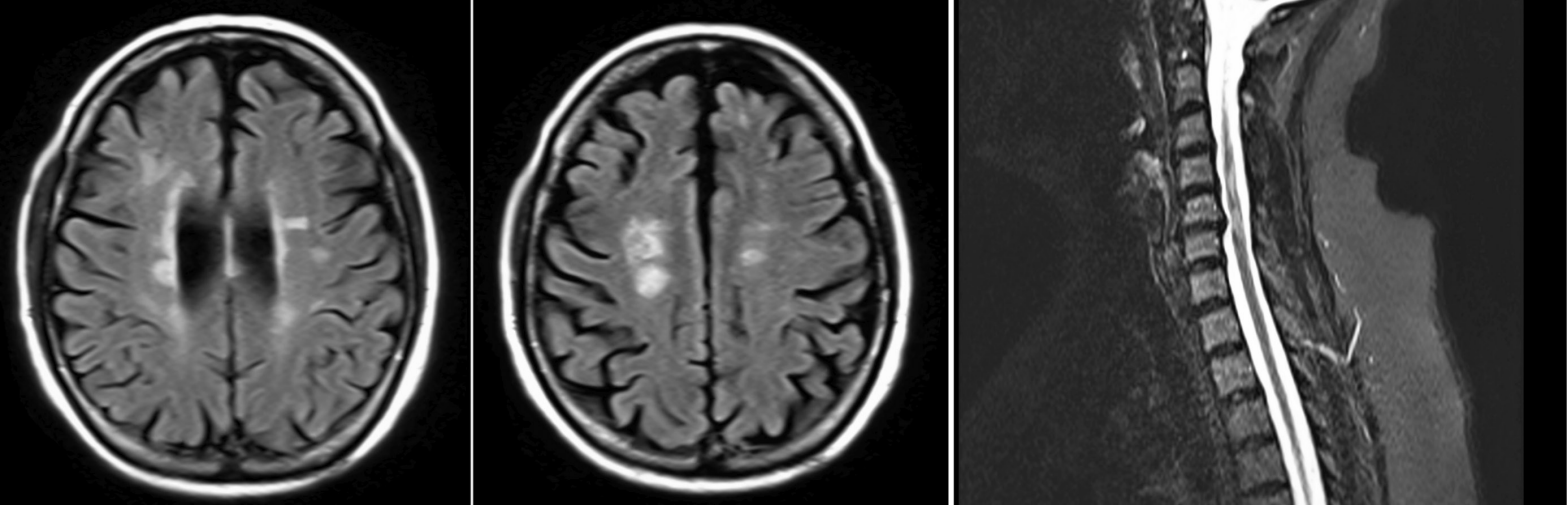
Case 1: fluid‐attenuated inversion recovery (FLAIR) MRI sequences showing high‐intensity areas in paraventricular and para‐spinal regions.

A 53‐year‐old woman presented to our clinic in March 2019 for evaluation of lower limb weaknesses along with visual impairments starting from 2 months earlier. She was married with three children, had a history of a face lesion 5 years prior, for which she had visited a dermatologist. At that time, she was diagnosed with sarcoidosis based on a histopathological study of the lesion biopsy which revealed a pattern of non‐caseating granulomatous inflammation consistent with sarcoidosis, a chest CT study which found mild pleural thickening and hilar prominence on the right side, and serum angiotensin‐converting enzyme (ACE) level measurement, which was within normal range but close to the upper limit. Since then, she was put on oral prednisolone therapy. From the time she was diagnosed with sarcoidosis, she reported experiencing several episodes of ataxia, diplopia, and upper limb weakness, each lasting between 2 and 4 weeks. Those episodes were deemed due to NS and treated accordingly in other clinics. She also developed hypertension and diabetes mellitus, otherwise, her past medical, social, and familial histories were unremarkable. Neurological examination revealed glove and stocking sensory impairment and lower limb weakness with a distal force of 3/5 and proximal force of 5/5. Babinski sign was observed bilaterally and tendon reflexes were brisk. Considering NS, diabetic neuropathy, and MS as the primary differential diagnoses, nerve conduction velocity (NCV) studies, visual‐evoked potential test (VEP), and MRI were performed. NCV showed demyelinating polyneuropathy in the lower extremities, VEP results were 192 and 194 in the left and right eye, respectively, and showed prolongation of latency bilaterally. MRI study showed multiple demyelinating lesions in the periventricular, juxtacortical, and cervical spine areas (Figure 1). Although some findings could have been explained by diabetic neuropathy and/or NS, the clinical course of the illness and the location and features of the lesions in MRI were consistent with, and typical of MS; therefore, in accordance with the McDonald criteria,^17^ diagnosis of relapsing–remitting MS was made. CSF analysis for detection of oligoclonal bands (OCB) was not performed, as the patient was non‐consensual for a lumbar puncture, and already fulfilled the criteria of dissemination in space and time. She was offered rituximab therapy but refused to undergo the treatment. Unfortunately, the patient died at the age of 54 reportedly due to myocardial infarction—6 months after visiting our clinic.

## 3 CASE PRESENTATION 2

**FIGURE 2:**
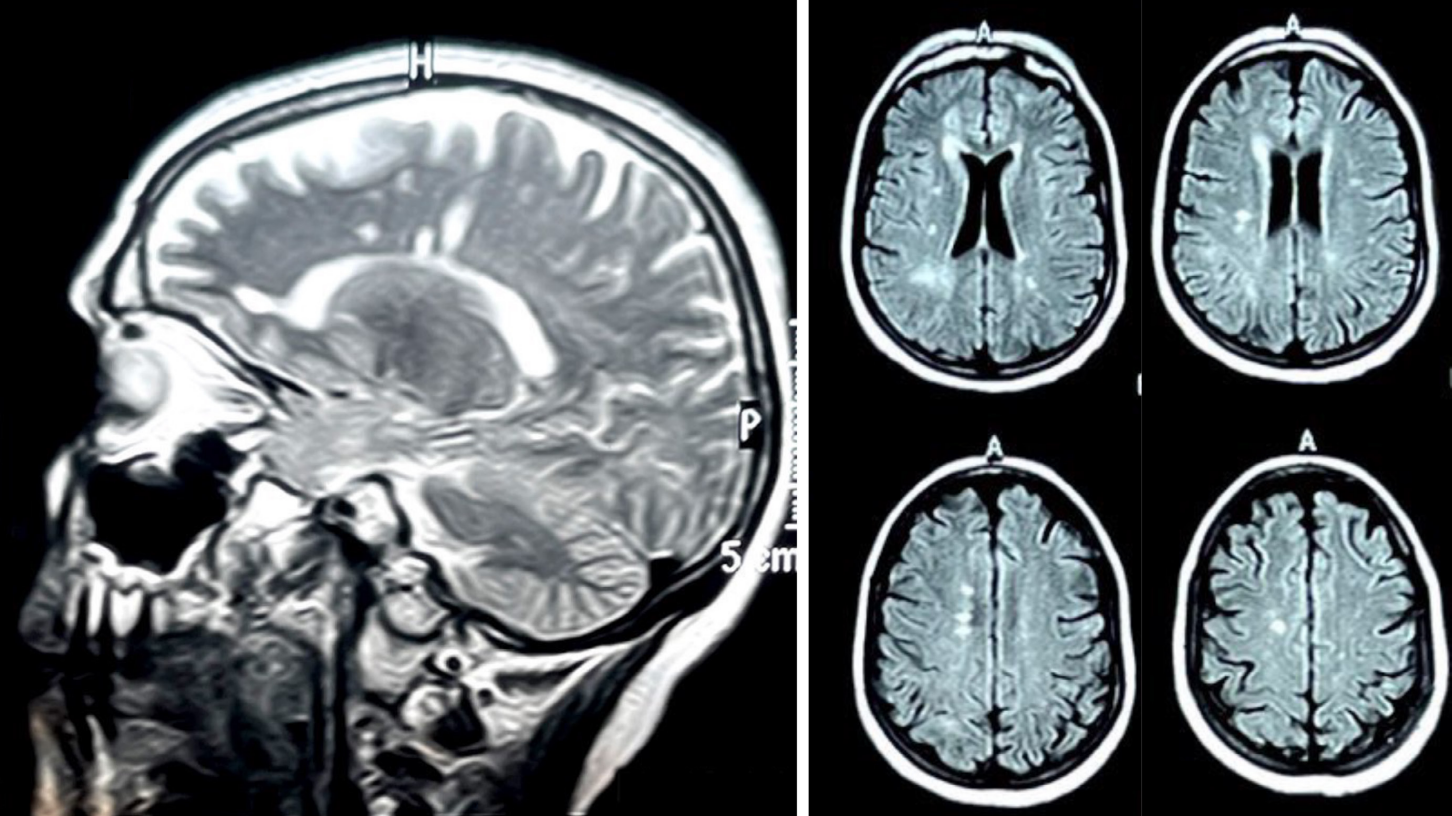
Case 2: sagittal T2‐weighted and axial FLAIR brain MRI sequences showing typical MS lesions in periventricular (Dawson fingers) and juxtacortical regions.

In July 2019, a 60‐year‐old woman presented to our clinic with lower limb weakness, paresthesia, and urinary incontinence. She was widowed with one child, had a past medical history of right facial paralysis 14 years prior diagnosed as Bell's palsy. Then, she started to develop intermittent inflammations in both knees, and also a sharp focal pain in the left side of the chest, which intensified with respiration. Subsequently, chest radiograph and CT scans showed left pleural thickening consistent with pleurisy, and small nodular lesions in the right lung. Pathological study of the lesion needle biopsy showed non‐caseating granulomatous inflammatory reaction, serum ACE was measured to be close to the upper limit of the normal range, and tuberculosis workups were negative. Back then, she was diagnosed with sarcoidosis, and was taking oral prednisolone ever since. She had been experiencing neurological episodes from time to time since then, each lasting a few weeks as she stated. For which, she visited a nearby clinic, was diagnosed with NS, and was treated accordingly. Other medical, social, and family histories were unremarkable. Neurological examination showed lower limb weakness and bilateral Babinski sign. No sensory level was apparent. Considering NS, tumors, and MS as the most probable diagnoses, MRI was performed, which showed cervical spine, periventricular (Dawson fingers), and juxtacortical demyelinating lesions suggestive of MS (Figure 2). As she also fulfilled the dissemination in time criterion, based on the McDonald criteria,^17^ she was diagnosed with MS without CSF analysis to determine the presence of OCBs. She was prescribed weekly Interferon beta‐1a injections which she complied with. After consulting a pulmonologist, adding methotrexate to the treatment plan was suggested and was done. In her last subsequent follow‐up in June 2022, she was in good condition with complete resolution of her lower limb weaknesses, no additional relapses, an expanded disability status scale score of 1.5, stable breathing with no dyspnea at rest, and no other major problems.

## 4 DISCUSSION

Sarcoidosis can produce several heterogenous clinical pictures, stemming from the multisystem nature of the disease. NS is uncommon, but also a potentially serious form of sarcoidosis. While NS mostly affects cranial nerves, it can also involve other parts of nervous system, including intracranial structures. The clinical manifestations in an individual depends on the location of the inflammatory process. The most commonly reported features of NS include cranial nerve neuropathy, headache, fatigue, nausea and vomiting, sensory abnormalities like visual disturbance, motor symptoms consisting of hemiparesis and paraparesis, meningitis, seizures, and spinal cord abnormalities.^18^, ^19^ NS can mimic a wide range of other neurological diseases such as neoplasms, infections, angiitis/vasculitis and demyelinating diseases—including MS.^20^, ^21^, ^22^, ^23^ For instance, Serrano and colleagues report a patient, in whom NS was initially misdiagnosed as probable MS.^24^ Their case did not fulfill the criterion of dissemination in time, and was therefore followed up on a regular basis until diagnosed with sarcoidosis in a subsequent workup for cough and dyspnea.^24^ Apart from the characteristics and location of the lesions,^14^ Serrano et al.’s case demonstrates the importance of the dissemination in time criterion in discriminating MS from NS—although NS could also show a relapsing–remitting clinical course. CSF OCB are also a powerful discriminator of MS from NS as suggested by Arun et al.,^25^ but they also may turn out positive in NS cases.^14^

Furthermore, although cases of sarcoidosis in people with preexisting MS—particularly deemed, but not proven to be associated with certain DMTs^14^, ^26^, ^27^, ^28^, ^29^, ^30^—and NS cases mimicking MS^23^, ^24^, ^31^, ^32^, ^33^, ^34^ have been frequently reported in the literature, few cases of MS have been reported in patients with preexisting sarcoidosis (Table 1). In known sarcoidosis patients who develop a chronic neurologic illness, “there is a tendency to assume that NS is the highest possibility” as stated by Tyshkov and colleagues.^14^ Remarkably, diagnosing MS in known sarcoidosis patients may be of a greater challenge than diagnosing sarcoidosis in known MS patients^35^—as unlike the former, subsequent development of sarcoidosis in the later cases could be detected and confirmed based on extra‐nervous findings. Furthermore, misdiagnosing MS as NS delays and further complicates a correct diagnosis of MS, as MS becomes less prevalent in higher ages—especially in ages above 50—and neurological illness at higher ages are less likely to be deemed because of MS; for example, our cases were diagnosed above the age of 50, but could not be classified as late‐onset MS as their symptoms developed before the age of 50. This might as well be the reason that the later has been more frequently reported in the literature. In presence of such challenges, a thorough neurological evaluation and determination of the true date of symptom onset, along with imaging, electrophysiological studies, and CSF analysis seem to be reasonable before marking sarcoidosis patients who develop a neurological illness as NS cases. Nevertheless, in cases of coexistence or uncertain diagnosis, a practical approach could be treatment options that benefit both conditions, for example, methotrexate therapy^14^, ^35^—as done in our second case with a favorable outcome after 3 years.

**TABLE 1:** Summary of studies reporting MS in patients with preexisting sarcoidosis

Reference (location)	Age upon diagnosis (years)	Initial presentation of sarcoidosis	Initial neurologic symptoms	MRI findings	Other findings	Method of final differentiation of MS from NS
^37^(Croatia)	MS: 49 Sarcoidosis: 32	LAD	Lower limb weakness and numbness	Demyelinating lesions in brain	Non‐specific laboratory findings, no information on presence of OCB in CSF	Treatment trials, clinical course
^14^(USA)	MS: 30 Sarcoidosis: 25	Löfgren syndrome	Hemifacial numbness	Demyelinating lesions in brain and spinal cord	Not specified	MS‐typical lesions, clinical course, CSF analysis
Present report (Iran)	MS: 53 Sarcoidosis: 48	Skin lesion	Gait ataxia, diplopia and upper limb weakness	Demyelinating lesions in brain and spinal cord	Slowed NCV in lower extremities, Latency prolongation of VEP in both eyes, no information on presence of OCB in CSF	Treatment trials, MS‐typical lesions, clinical course
Present report (Iran)	MS: 60 Sarcoidosis: 46	Pleurisy, knee inflammation	Lower limb weakness, and gait ataxia	Demyelinating lesions in brain and spinal cord	Non‐specific laboratory findings, no information on presence of OCB in CSF	Treatment trials, MS‐typical lesions, clinical course

Abbreviations: CSF, cerebrospinal fluid; LAD, lymphadenopathy; MRI, magnetic resonance imaging; MS, multiple sclerosis; NCV, nerve conduction velocity; NS, neurosarcoidosis; OCB, oligoclonal bands; VEP, visual‐evoked potentials.

Finally, no underlying mechanistic correlation between sarcoidosis and MS has been confirmed to date, although both are thought to involve genetic and immune‐mediated etiologies. Although not established, the HLA loci may be involved in both MS and sarcoidosis.^36^ Further studies may provide clues to any common genetic links between the two diseases and provide useful guidance regarding disease pathology and effective therapy.

## 5 CONCLUSION

Differentiating MS from NS may be challenging in known cases of sarcoidosis. We reported our experience with two cases of sarcoidosis patients developing MS, who were diagnosed and managed without any major invasive procedures. This report, along with the previous ones, provides valuable insights on the diagnostic work‐up of neurological symptoms in sarcoidosis patients.

## AUTHOR CONTRIBUTIONS

PTN, ME, and AM have gathered relevant case information, searched the databases for relevant data, and drafted the initial manuscript. MS and NS were involved in validation of findings, review, and editing of the initial draft. All authors have read the final manuscript and approved it for publication.

## CONFLICTS OF INTEREST

The authors have no conflicts of interest to declare.

## CONSENT

The patients or their next of kin provided written consent for publication of their anonymized cases.

## Data Availability

No other data from what is reported was gathered/generated.
